# Values exchange: using online technology to raise awareness of values and ethics in radiography education

**DOI:** 10.1002/jmrs.258

**Published:** 2018-02-01

**Authors:** John Mc Inerney, Amanda Lees

**Affiliations:** ^1^ Monash University Melbourne Australia; ^2^ Auckland University of Technology Auckland New Zealand

**Keywords:** Clinical‐decision making, educational technology, ethical‐decision making, professional practice, student

## Abstract

**Introduction:**

Ethics and values are increasingly significant aspects of patient‐centred healthcare. While it is widely agreed that ethics and values are essential for healthcare delivery, there is also an acknowledgement that these are areas that are challenging to teach. The purpose of this study is to report a small‐scale evaluative research project of a web‐based technology with the educational potential to facilitate learning in relation to ethics, values, self‐reflection and peer‐based learning.

**Methods:**

Five diagnostic radiography students took part in a semi‐structured focus group with the aim of exploring their experiences of using Values Exchange, an online ethical decision‐making framework, to examine practice‐based ethical issues. Transcripts were interrogated for key themes.

**Results:**

From the thematic analysis three major themes emerged, understanding and appreciating others, addressing the theory‐practice gap and delivering a safe and effective learning environment.

Perceived limitations of the platform included students' fear of misinterpreted responses and possibility of poor group dynamics.

**Conclusions:**

There are varied approaches to how ethics and values are taught and assessed within health‐related environments. Values Exchange is one such teaching tool and has been investigated and described positively by radiography students in this study. Online teaching tools can have a positive effect in helping students identify their own values but require skilled implementation to reap positive rewards.

## Introduction

Adherence to ethical standards are requirements for registered health professionals and radiographers are no exception.^1^, ^2^, ^3^, ^4^, ^5^, ^6^, ^7^, ^8^ Given the pressured space within curricula, teaching activities, proven effective for equipping health professionals with such skills, must be prioritised.

Ethics and values are increasingly significant aspects of practice as trends shift towards patient‐centred healthcare.^9^, ^10^ With requirements for radiography practice surpassing content knowledge and competency in practical skills,^11^ radiographers are obliged by professional bodies and registration authorities to embrace contemporary models of competency, including ethical practice.^4^, ^5^, ^6^, ^7^ Health professionals need to be critically reflective and challenge existing pre‐suppositions. Issues of professional practice need to be examined, including ethics and morals.^12^ Lewis et al^13^ argue that Australian radiographers attempting to set standards of ethical commitment are hindered by medical dominance, low professional independence and difficulty accepting responsibility. It has been suggested that private radiology business models can erode radiographer‐patient relationships and have the potential to introduce unethical practice in the radiographer‐radiologist‐referrer relationship.^13^ Some evidence suggests that ethical violations are increasing within radiography.^14^

Ethics and values are challenging to teach.^9^, ^10^ Teaching radiography traditionally involves lectures, traditional simulation and clinical placements, however the connection between science and clinical decision‐making has been questioned in using these approaches alone.^11^ Godbold & Lees^9^ describe two approaches to ethics education; outcome‐based ethics, where responses are objectively measured against predetermined responses, and process‐oriented ethics, which recognises the subjective nature of ethical situations and facilitates self‐reflection and critical analysis. Increasingly the literature supports the latter with increased focus on understanding the role of values in decision making and less on learning to ‘do the right thing’.^10^, ^15^ Ethics education ought to help students identify their own values and decision‐making processes rather than instilling specific beliefs.^16^ Brookfield suggests that if students identify their own assumptions and challenge them, they will be more capable critical thinkers.^17^ Research by Shinyashiki et al^18^ suggest that universities overlook the necessary exploration of incumbent students' values to assume professional roles in clinical placements and beyond.

Online learning environments (OLEs) offer flexibility for users and the ability for students and teachers to adopt non‐traditional roles and for student‐centred learning to flourish.^19^ However, Luke et al^20^ suggest that OLEs are sometimes seen as a solution to time and location constraints. Thus, educators are challenged to develop innovative teaching curricula that promote self‐reflection and student‐centred learning.^21^ Evaluation of online teaching methods is therefore essential to inform practice and ensure students are suitably equipped for their professional roles.

This paper reported on radiography students' experiences of a web‐based technology to facilitate learning in relation to ethics, values, self‐reflection and peer‐based learning.

## Methods

Values‐Exchange (Vx) is an OLE designed to facilitate ethical decision‐making and values transparency.^10^ Users, academic staff or students, can post ethical dilemmas and case scenarios for shared exploration, within a wider social networking framework. In this instance it is the academic staff who post the dilemmas for the students to consider.

**Figure 1:**
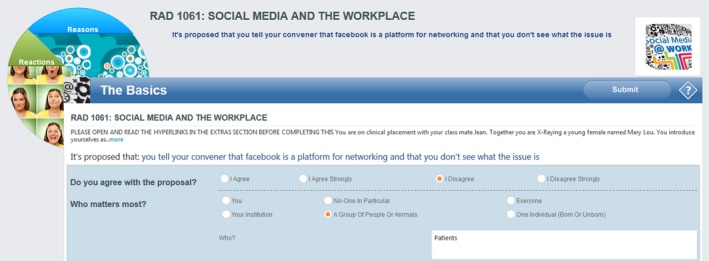
Think screen #1, The basics.

**Figure 2:**
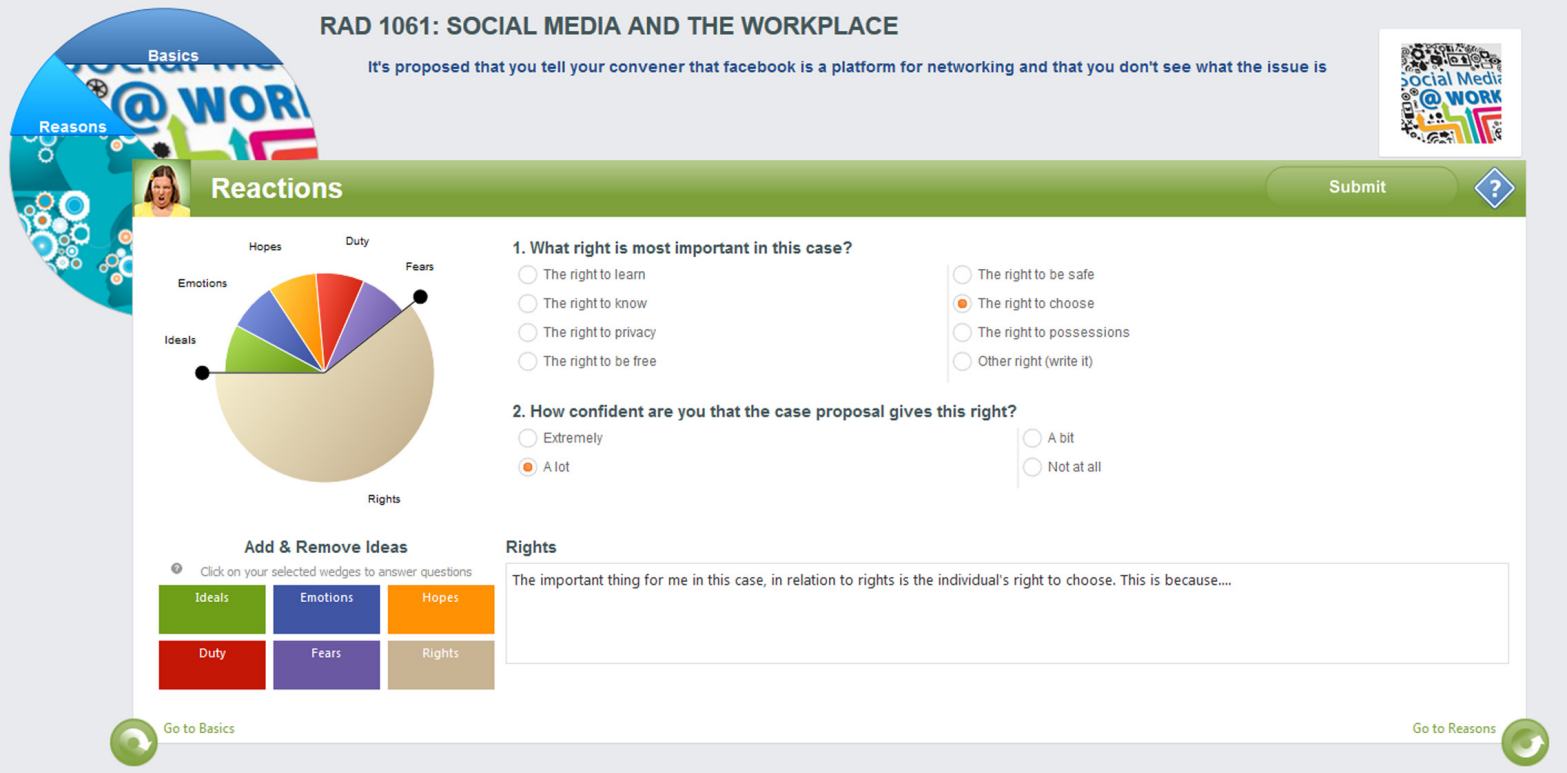
Think screen #2. Reactions.

**Figure 3:**
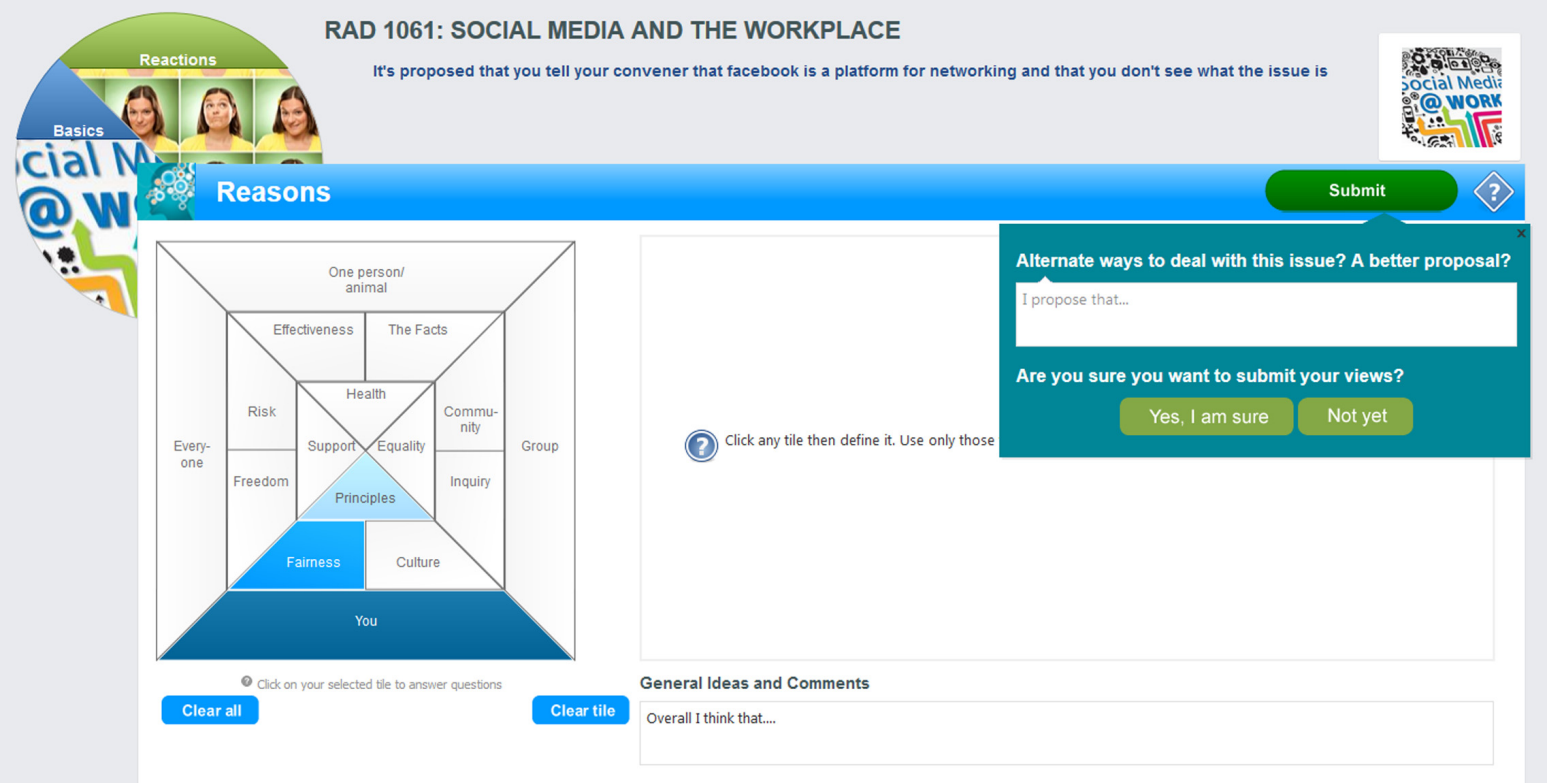
Think screen #3. Reasons, including alternative proposal.

Questions are posed in a number of formats including polls, surveys and the unique, theory‐based, Think Screen (Figs. 1, 2 and 3), each utilising a proposal as the basis of deliberation, with no definitive ‘right’ answer. Unlike methods where specific answers are expected, responses in Vx are assessed in terms of the strength of rationale, in keeping with a process‐orientated method of ethics education.

Based on philosophical work by Seedhouse,^10^ the Think Screens (Figs. 1, 2 and 3) guide users through a deliberative decision‐making process. It uses an analytical framework based on elements of traditional ethical theory, such as duty and benefit, as well as practical considerations such as evidence and the environment.

In the Basics Screen students indicate their level of agreement with the case proposal and consider who matters most in the scenario (Fig. 1). Followed by the Reactions and Reasons screens (Figs. 2 and 3) students must consider a range of perspectives and ethical considerations, providing reasoning to support choices made. Cases posted include exploration of issues such as the role of social media, role definitions and cultural awareness (Table 1).

**Table 1:** Synopsis of cases posted in values exchange

Case 1 – ‘THAT'S NOT MY JOB’: You are on an ED rotation when a sick patient arrives who has dementia. While they are waiting in the trolley bay they vomit on themselves and their bedclothes. The radiographer tells you they will not go near the patient until they are cleaned up by nursing staff. In this case, students must identify the professional issues. This is a cross‐discipline case that was developed in conjunction with the Department of Nursing and Midwifery. In the tutorial we review responses from the nursing perspective to gain insight into another discipline's perspective comparing them to radiography students. Case 2 – ‘OVERLY ANXIOUS ELDERLY’: An elderly male is accompanied by his wife and son to the X‐Ray Department. The son is the only English speaker. The family gets upset when the radiographer refuses the family entry to the room despite the upset it will cause. Case 3 – ‘SOCIAL MEDIA AND THE WORKPLACE’: You and another student Radiographer use a workplace computer to access Facebook. You use the request form of a patient you X‐rayed earlier that day to find their details and send them a Facebook message asking for their assistance securing employment. You can't understand the implications of your actions when the person complains to the University about the invasion of their privacy. Case 4 – ‘POTENTIAL EXPOSURE’: You are on your very first placement; one of the radiographers, who is pregnant, is holding a difficult patient and asks you to ‘prep’ the tube until she is ready to get behind the screen. You are not sure what she means as you have not used this machinery before. Do you decide to try and figure it out? Case 5 – ‘CULTURALLY COMPETENT?’ A widowed Muslim woman is recovering from a hip operation. Her extremely devout son is upset that she is being cared for by males and is insistent that male health workers cease treating his mother. Students explore whether they feel that male health professionals should remain a part of the team caring for his mother no matter what. This is an extended roleplay case. The students must explore the case at their own time and come prepared to take part in the roleplay in a small group (5 students per group) tutorial.

The choice of the Vx as a teaching tool reflects the pedagogical practices valued within the Monash University radiography programme. It allows a blended approach to learning and enables students to be active in the learning process.

**Figure 4:**
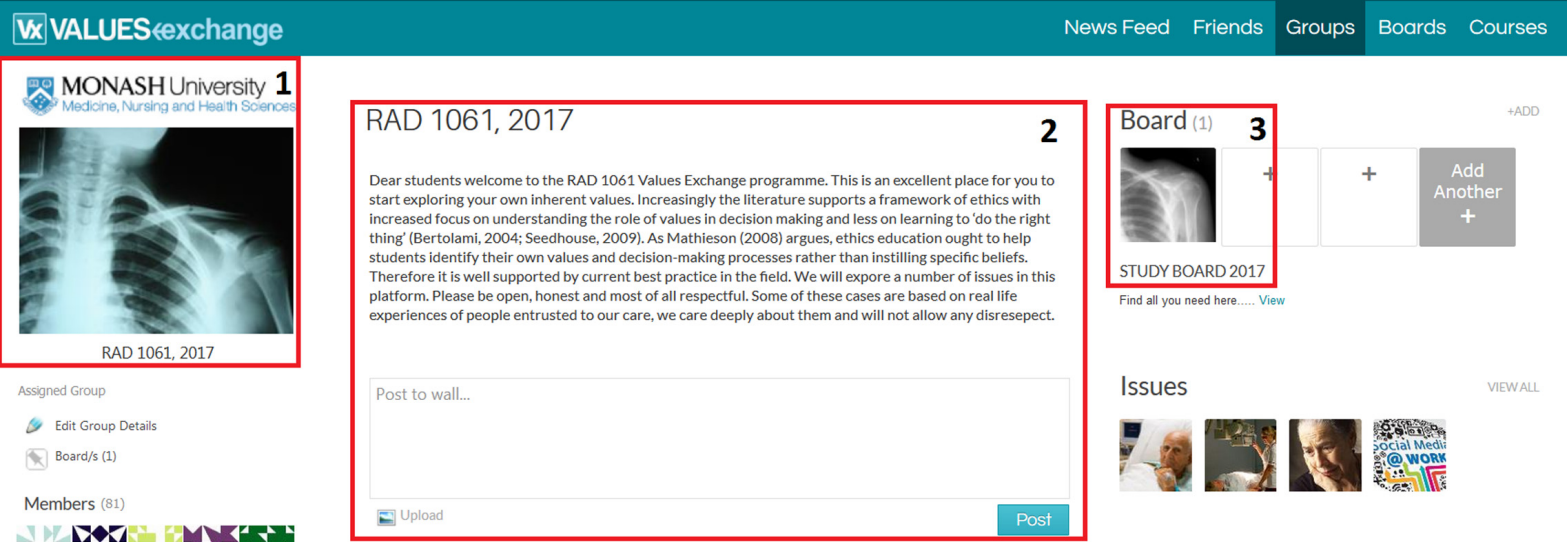
Monash University Radiography group Vx dashboard.

**Figure 5:**
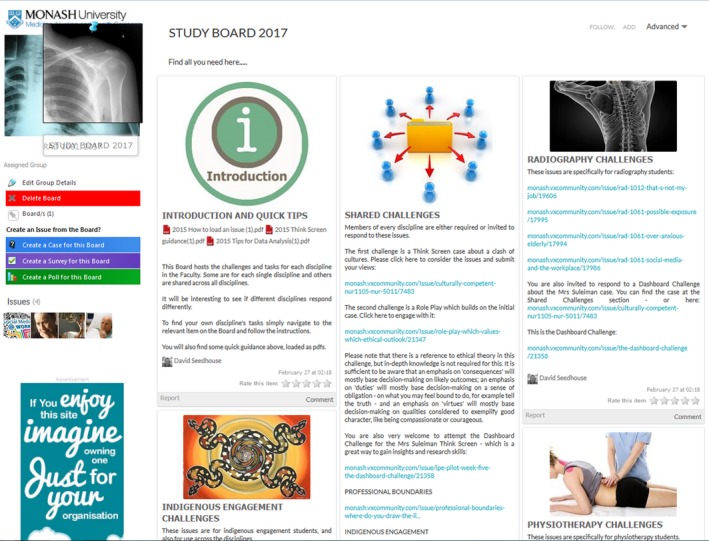
Monash University Faculty of Medicine Nursing and Health Sciences Vx Shared Board.

Vx is introduced in semester one of first year and students engage with the platform throughout the semester. The course is an undergraduate course and in this semester students focus on developing foundational radiography‐based knowledge.^11^ Vx allows students to spend some time preparing for other vagaries of clinical placement. A message ‘wall’ allows lecturers to post announcements (Fig. 4, #2) and there is a link to a faculty shared board (Fig. 5).

Some are radiography‐specific, some are ‘shared’ across disciplines. These are accessed by radiography students as well as disciplines including medicine and nursing. There is also an indigenous engagement section.

The study was exploratory and interpretive. There is limited literature on values and ethics in radiography education and the use of Vx as a teaching tool had not been studied. Exploratory methodology is appropriate where existing knowledge is limited.^22^ A 90 min semi structured focus group was conducted with participants’ conversations guiding the discussion. Focus groups are an appropriate method for exploratory research.^23^

First year diagnostic radiography students enrolled in the unit in 2015 (*n* = 75) were invited to participate by contacting the lecturer. This was augmented with a snowballing recruitment method, with students advising peers of the opportunity, which can broaden the reach of the research.^24^ Five students were recruited to participate and the focus groups took place in July 2015, constituting a 3 months gap between the students participating in the Vx course and the data collection.

Ethics approval granted by Monash University Human Research and Ethics Committee (MUHREC), project number CF15/1681 – 2015000844. An explanatory letter in plain English ensured informed consent and outlined the complaints procedure for students. The transcripts were anonymised and transcribed by a third party to ensure anonymity. Participation was voluntary and students were free to withdraw at any stage. Since the investigators were also involved in the assessment of the subject for which this intervention was implemented, it was stipulated in the ethics approval that the research participants could not be recruited until the unit was entirely completed and all results published to the students.

The focus group transcript was examined through a process of thematic analysis informed by Braun & Clarke's^25^ six‐step method. Each researcher independently familiarised themselves with the data, making initial codes, followed by searching and reviewing themes. After defining and naming themes, each analysis was examined by both researchers for commonality. Three themes were identified through shared consideration and synthesis.

## Results and Discussion

While it might be said that a learning activity can only be reported as achieving its goals if the students undertake pre‐ and post‐testing to demonstrate discernible change, it has been previously argued that students' perceptions are a valid indicator of attitudinal change from engaging with the activity.^26^

### Theme 1: Understanding and appreciating others

Brookfield^16^ describes the importance of generating multiple viewpoints for consideration by students and believes this is best achieved in group learning. It appeared that Vx offered an opportunity to learn from, and about, others. Students reported that Vx afforded the opportunity to learn about the pressures others are under, which may not be overt. One participant noted that the Vx role play facilitated ‘getting into different shoes’… ‘if you get an angry patient you don't know what their circumstances are, initially from our perspective we'll perceive it as a patient who's quite aggressive and angry. You've got to kind of go beyond that and go you know he's been through a lot of things’ (Student A).

While the importance of interdisciplinary teamwork is widely acknowledged as being important in delivering health care and important in providing care for older patients, Leipzig et al^27^ suggest that this is rarely a focus in the education of health professionals. Students in this study felt they learnt about other professionals within radiography and other disciplines. ‘Other goals exist that [are] parallel with yours so at times as a radiographer your goal is to get the best images possible based on your patients situation and you have to learn that other people's goals are just as important even though it's entirely different from yours’ (Student C). ‘There's a bigger community outside than just the radiographer and the patient…we've got to do our part as well’ (Student A).

Furthermore, as student B highlighted, awareness of teamwork can lead to positive patient outcomes. ‘[There are] many people just helping that one patient alone. We might have one way of thinking but there's also other people that have other ways of thinking too that all together will give the patient the best patient care in the end’(Student B). Clark et al^28^ argue that having an understanding of how one's own discipline and others work and how each views the patient are important components for practicing critically and ethically within a teamwork environment.

One participant felt that using Vx could ‘relieve some of the tension that you get in the workforce’ by allowing students and practitioners to ‘communicate across each domain and to criticise each other in a safe zone it would help for understanding their point of view, their duties and what they have to do’ (Student B).

All students acknowledged the importance of accommodating the values and perspectives of others. Student C commented that one might ‘have something to say to begin with and then you start to change as you start to hear a few different things as well. I think that's another benefit from Vx as well’. One student provided an insightful reminder that while ‘it's important to acknowledge other people will make a decision, but also if you think you're correct you should stick by that as well. I think that's very important’ (Student A).

Shinyashiki et al.,^18^ reporting on nursing students entering college, acknowledged that students have pre‐existing values which may be contrary to the objectives of the profession. Students require guidance to change their values through a ‘socialisation process’^18^ ^,p 602^ to reflect those of their profession. While more research is needed to fully understand values and how they guide our practice‐based decisions,^29^ there was some evidence that Vx helped students understand that their different backgrounds necessitate due consideration when exploring their values and the decisions they make. ‘We are different age, different nationality so we have to respect different situations’ (Student D). This mirrors findings from previous Vx studies, with participant groups representing those found within the radiographers interdisciplinary team environment, such as nursing and physiotherapists.^9^, ^30^, ^31^

### Theme 2: Addressing the theory‐practice gap

Transferring theory to clinical environments has been the subject of considerable literature. Clinical placements can be stressful, the change in the learning environment causes anxiety for students and can negatively impact learning.^32^ ‘You've got a lot to learn on placement to begin with’ Student A.

Vx encapsulates learning activities that can contribute to students’ readiness to practice. Case‐based learning provides an opportunity for students to integrate theoretical knowledge with their experiences on placement, addressing the theory‐practice gap.^33^ Since Vx is a case‐based platform used to explore professional scenarios and skills, it has the potential to bridge the chasm between theoretical evidence and its application in practice. Participants echoed this and felt Vx went some way towards building confidence for clinical placement and transferring theoretical aspects into a reality by giving them a ‘bit of a heads up’ before attending placement (Student A). Student A continued by suggesting that Vx provided students with ‘some idea of how to approach patients… that's pretty important’. Student C reported that Vx ‘gives you that bit of confidence to go out to the real world…it gives you a rough idea what you can expect’.

Students' responses suggested that the activities associated with Vx gave them increased confidence in values‐based decision‐making in complex situations. ‘I think it gives you confidence and like experience to deal with when you're in those situations… whereas I think if you went on to placement with no idea how to handle people… you'll probably shy away and let the supervisor deal with it’ (Student B).

### Theme 3: Delivering safe and effective learning

A key feature of student‐centred learning is that students must feel safe within the learning environment so they can take responsibility for their learning.^34^ Students agreed that Vx provides this. Student E noted that ‘I think that's important. It's OK to be scared here cos it doesn't matter how you respond to it but when you get to placement you don't want to be scared…it's prepared us’. Student B noted that ‘we're not scared to say our views so it keeps us open minded for the future’. This suggests that while Vx allows students to air their views, they remained cognisant of the fact that they may equally change their perceptions as they mature and have new experiences. Student A said that Vx allows them to ‘test that what you think is right’ as it ‘doesn't have that many consequences compared to outside’.

There was a sense that students considered Vx a shared learning experience and that this enhanced the learning experience. ‘If we all talk to each other or discuss with each other we feel more prepared to go out into the real world’ (Student D). Student E felt that since ‘every‐one's contributing I feel like you're not putting as much effort but you're getting more out of it’ this student also felt that because of the group work it felt like they were ‘getting the feedback on how you tackle a situation’. Student A suggested that due to the group dynamic ‘you start thinking OK, that's another alternative, based on this situation’ and you are therefore ‘learning from your peers as well…..kind of fine tuning your opinion’. They were mindful of the fact that ‘it's the best way to learn as well because if you're doing it by yourself you're thinking one minded’. Student A stated that ‘we're not going to be exposed to the same scenario… let's say I don't get a situation that's like that, I could obviously learn from other people's experiences as well and incorporate that so that when I do encounter something like that I've got some idea of how to manage it’.

## Implications of the Research

According to Kirkpatrick & Kirkpatrick^35^ a key reason for evaluating learning activities is to provide direction for future improvements. The students provided insightful commentary for future development of Vx.

Several students commented that the inter‐professional aspect of the platform could be developed further. It is encouraging that students have noted this potential from engaging with the platform as further development of Vx in Interprofessional Education (IPE) is already underway. Student C reported that ‘You see the same radiographers every day that you're working’ but that ‘some of the interprofessional stuff would be good’. Student D suggested that if ‘different responses from different professionals’ were recorded this would allow ‘for people who have come from the real world [qualified staff from various disciplines] to come up with answers and so we can think openly about different perspectives’.

Student B remarked that Vx is a platform in which reflection over time can occur. ‘Your answers are safe so if you were to go back in a few years and look back and reflect on it and think; I would've done something differently’. Student E said that as a student one is encouraged and made to reflect as part of their academic requirements but constraints such as time pressures mean qualified radiographers may not reflect as effectively. They mention that Vx would allow one to ‘reflect on yourself and where you are in a few years and see how much you've changed’. This is a profound observation given that only in 2013 the MRPBA, in describing the professional attributes necessary registration for entry level practice, moved away from a ‘competency’ based framework to a ‘capability’ framework, acknowledging the new dynamic in health care requiring practitioners to utilise reflective practice to resolve clinical dilemmas.^4^

For future research the authors aim to appraise if engaging with the values exchange programme served to enhance students’ emotional intelligence using a before and after validated tool to measure this.

## Limitations of Vx

While the overall Vx experience of the participants was positive the students did highlight perceived limitations of using Vx.

Student B said in regard the tiles (Figs. 1, 2, 3) that ‘I wasn't sure what I was meant to do with it, like should I click every single one and answer every single one or just the one I wanted and I was just like I don't like that part’. This is possibly due to the limited time students had to truly get to know the platform well. However this could likely be addressed with some simple instructional changes, preferably in the Vx platform itself. Chow et al^36^ stress the importance of adequate training of end users in computer environments to enhance implementation.

Bourner^37^ suggests that something which is not assessed will likely be neglected. Despite the fact that these students are extremely capable and driven the question ‘why do I need to do this, is it compulsory?’ and the answer ‘if it's not compulsory then I'm not going to do it’ was raised by Student B. Despite this perception however, when an analytical report was conducted, the rate of participation in the Vx programme was high.

Student C feared that while Vx exposed them to multiple viewpoints, how people interpret their perspective might be problematic as ‘you can't get feedback on your view’ or take the interpretation and modify how to express your views to make them understandable to others.

## Limitations of the Research

There was a 3 months gap between the Vx activities and the focus groups due to clinical placement and a 3 weeks University holiday. It was a stipulation for ethics approval that students would no longer be enrolled in the unit in which Vx was used to avoid any conflict of interest. However this consequently allowing us to gain insight into the ability of Vx to transcend the ‘theory/practice gap’ with students having attended clinical placement before the focus group.

While low participant numbers was a limitation the discussion was in‐depth. The students reflected homogeneity, in regards class membership, but varied in their backgrounds and reflections. Stalmeijer et al.^22^ propose that ‘the advantage of group homogeneity is the familiarity that comes from shared background or experiences which can go a long way in facilitating open communication and exchange of ideas’. This was the experience in this study.

## Conclusion

Ethics and values are increasingly significant aspects of practice as patient‐centred healthcare becomes the norm. At the same time online learning has gathered pace. Therefore, the challenge for educators is to interweave the two such that one is implemented effectively to leverage the other with good outcomes for students and ultimately, patients. New approaches to teaching, learning and assessment are imperative to improve the student experience. From the study Vx appears to have had a net positive effect with students reporting increasing confidence in decision making in real‐life clinical situations. This coupled with the opportunity to develop Vx as an IPE tool and opportunities for critical self‐reflection suggest that the Vx offers a unique and valuable tool to achieve teaching and learning goals.

## Conflict of Interest

The authors declare no conflict of interest.
